# Noninvasive Retinal Markers in Diabetic Retinopathy: Advancing from Bench towards Bedside

**DOI:** 10.1155/2017/2562759

**Published:** 2017-04-13

**Authors:** Søren Leer Blindbæk, Thomas Lee Torp, Kristian Lundberg, Kerstin Soelberg, Anna Stage Vergmann, Christina Døfler Poulsen, Ulrik Frydkjaer-Olsen, Rebecca Broe, Malin Lundberg Rasmussen, Jimmi Wied, Majbrit Lind, Anders Højslet Vestergaard, Tunde Peto, Jakob Grauslund

**Affiliations:** ^1^Department of Ophthalmology, Odense University Hospital, Odense, Denmark; ^2^Department of Clinical Research, University of Southern Denmark, Odense, Denmark; ^3^Department of Neurology, Kolding Hospital, Hospital Lillebaelt, Kolding, Denmark; ^4^Department of Regional Health Research, University of Southern Denmark, Odense, Denmark; ^5^Institute of Molecular Medicine, University of Southern Denmark, Odense, Denmark; ^6^Odense Patient Data Explorative Network (OPEN), Odense University Hospital and Department of Clinical Research, University of Southern Denmark, Odense, Denmark; ^7^NIHR Biomedical Research Centre at Moorfields Eye Hospital NHS Foundation Trust and UCL Institute of Ophthalmology, London, UK; ^8^Queen's University Belfast, Belfast, UK

## Abstract

The retinal vascular system is the only part of the human body available for direct, in vivo inspection. Noninvasive retinal markers are important to identity patients in risk of sight-threatening diabetic retinopathy. Studies have correlated structural features like retinal vascular caliber and fractals with micro- and macrovascular dysfunction in diabetes. Likewise, the retinal metabolism can be evaluated by retinal oximetry, and higher retinal venular oxygen saturation has been demonstrated in patients with diabetic retinopathy. So far, most studies have been cross-sectional, but these can only disclose associations and are not able to separate cause from effect or to establish the predictive value of retinal vascular dysfunction with respect to long-term complications. Likewise, retinal markers have not been investigated as markers of treatment outcome in patients with proliferative diabetic retinopathy and diabetic macular edema. The Department of Ophthalmology at Odense University Hospital, Denmark, has a strong tradition of studying the retinal microvasculature in diabetic retinopathy. In the present paper, we demonstrate the importance of the retinal vasculature not only as predictors of long-term microvasculopathy but also as markers of treatment outcome in sight-threatening diabetic retinopathy in well-established population-based cohorts of patients with diabetes.

## 1. Introduction

Diabetic retinopathy (DR) is the leading cause of blindness in the working-age population around the world [1, 2]. Given that diabetes is a global epidemic, we expect an increasing burden on the ophthalmological health care system for the upcoming years. Pivotal studies like the Diabetes Control and Complication Trial (DCCT) have demonstrated the beneficial effect of strict glycemic control in order to prevent or at least delay DR [3]. Nevertheless, the study also revealed that the combined effect of diabetes duration and glycemic regulation was only able to explain 11% of the total variation in the risk of DR, which leaves 89% for other reasons and mechanisms [4]. In addition, it took more than two years of strict glycemic control to demonstrate a beneficial effect on DR incidence and DR progression [3]. Hence, it is important to identify other mechanisms and early noninvasive markers of disease activity in order to stratify screening and treatment regimen for patients with DR.

The aim of this paper was to demonstrate noninvasive structural and metabolic retinal markers in patients with DR in particular as given by the evolving research at the Department of Ophthalmology at Odense University Hospital, Denmark.

## 2. The Retinal Vasculature

The theory of the principle of minimum work in the human circulatory system was presented by Cecil D. Murray in 1926 [5]. He hypothesized that blood vessels have an optimal vascular structure, which ensures sufficient blood flow with the least possible energy. This optimal geometry is perfectly adapted for the metabolic need. Deviations from an optimal vasculature will cause less efficient circulation and interrupt the metabolic capacity. This is in particular evident in diabetes which is known to be associated with changes in the optimal retinal vascular architecture [6].

Semiautomatic software has enabled in vivo, noninvasive evaluation of the geometrical features of the retinal vascular tree, hence providing valuable information about the condition of the systemic microvascular network and the impact of diabetes.

## 3. Retinal Vascular Fractal Dimension

Retinal vascular fractal analysis quantifies the fractal pattern of the retinal vascular tree [7]. Fractal patterns are a common phenomenon in nature and are seen in branching structures as frost crystals, tree branches, and lightning. Fractal structures are characterized by a self-similar pattern that is unaffected by difference in size. That is, under a different magnification, a smaller part of the whole will have the same structure as the bigger part. Retinal vascular fractal analysis is a validated method to describe the density and complexity of the retinal vascular tree in one parameter: the retinal vascular fractal dimension (Fd) [8]. Fd is defined as a noninteger unit between 1 and 2 (i.e., 1.4263), which increases correspondingly to the density of the retinal vascular tree.

**Figure 1:**
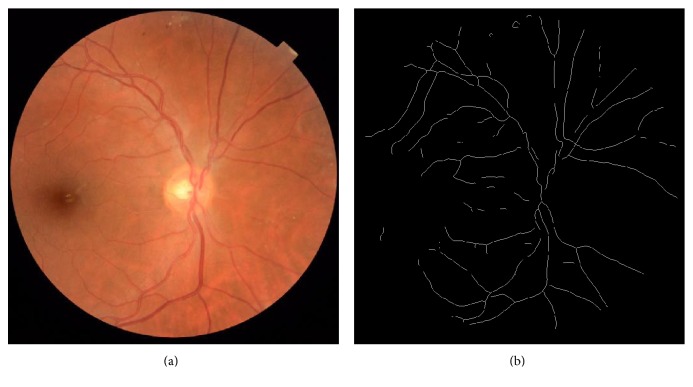
Cropped retinal image (a) and the corresponding skeletonized image (b) where all vessels are traced. Processed by fractal analyzer.

Semiautomated computer software like SIVA-Fractal (Singapore Institute Vessel Assessment-Fractal, School of Computing, National University of Singapore, Singapore) can be used to calculate Fd. Disc-centered color fundus photos are analyzed by a standardized protocol. The optic disc is automatically detected, and a grid is used to identify a zone between 0.5 and 2.0 disc diameters from the disc margin (Figure 1). All vessels that pass through the defined zone are automatically traced by the software which provides a skeletonized pattern of traced vessels. Any artefacts or misidentified vessels are manually removed by comparison to the original color fundus photo. Fd is then calculated by the software from the refined line tracing with the box-counting method. This is a well-defined method with a high intragrader reproducibility [8].

Previous studies have correlated lower retinal Fd with ischemic stroke, hypertension, and chronic kidney disease. In patients with diabetes, Fd has been correlated to vascular parameters such as retinal vessel calibers, blood flow, presence and progression of DR, and the development of proliferative diabetic retinopathy (PDR) [6, 9–14].

## 4. Retinal Vessel Caliber and Advanced Geometrical Measurements

Retinal vessel caliber can also be measured noninvasively [15, 16]. Several studies found a correlation between retinal vessel calibers and micro- and macrovascular complications in patients with diabetes [17–21]. Cross-sectional studies have associated wider retinal venular diameters with the presence of DR [6], and narrower arteriolar calibers have been correlated with more severe DR [22–25]. Prospective studies have also confirmed associations between wider venular calibers, progression of DR, and incident proliferative DR [26, 27]. In addition, two studies found wider arteriolar calibers as predictors for incident DR [28, 29].

Semiautomated computer software (IVAN, Department of Ophthalmology and Visual Science, University of Wisconsin, Madison, WI, USA) can be used to measure retinal vessel calibers in optic disc-centered color fundus photos. The optic disc is automatically detected, and a grid placed on top identifies a zone between 0.5 and 1.0 disc diameters from the disc margin. Vessels that course through this zone are automatically traced and marked as arterioles (red) or venules (blue) and manually adjusted for any inaccuracies in grid placement or tracing. Central retinal arteriolar and venular equivalents (CRAE and CRVE) are then calculated using the Big-6 formula. The method combines the diameter measures of the six largest arterioles and venules to estimate the diameter of the central retinal artery and vein which is then expressed as CRAE and CRVE, respectively [16, 30] (Figure 4(a)).

**Figure 2:**
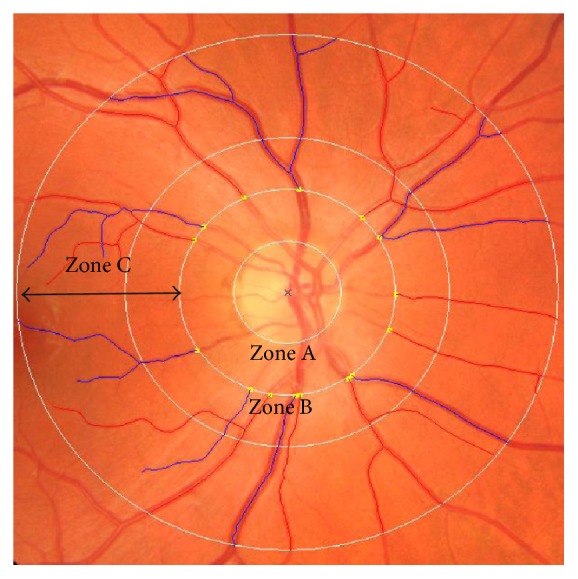
Retinal image processed by SIVA and used for analysis of vascular caliber, fractal dimension, tortuosity, length-diameter ratio, and branching coefficients. Marked are zone A, zone B, and zone C, located 0–0.5, 0.5–1.0, and 0.5–2.0 disc diameters from the center of the disc. In zones B and C, retinal arterioles have been marked in red and venules in blue.

In recent updates, measurements of retinal vessel diameter have been integrated in SIVA, where they can be performed in addition to measurements of advanced retinal vascular parameters like tortuosity (Figure 2), branching coefficient, and length-diameter ratio of the retinal vessels [19, 31, 32].

## 5. Retinal Oximetry

**Figure 3:**
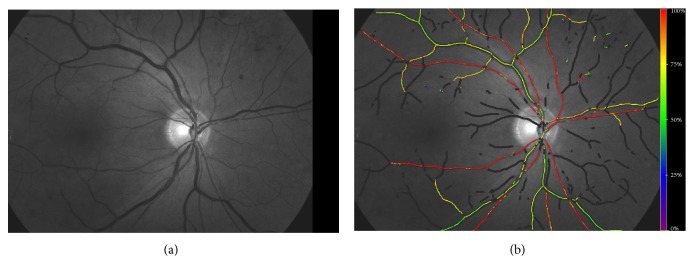
(a) Retinal image processed by Oxymap. (b) Vessels colored in accordance with the level of oxygen in the vessel.

Retinal oxygen metabolism has a key role in ischemic retinal diseases like DR [33–35]. The retinal oxygen saturation can be measured noninvasively in the larger retinal vessels by retinal oximetry based on dual-wavelength fundus photography [36]. In retinal oximetry, the oxygen saturation is measured by the color of hemoglobin given that oxygenated and deoxygenated hemoglobin has different colors. Retinal images of the same area are captured at a wavelength sensitive (nonisosbestic) and insensitive (isosbestic) to changes in absorptivity between oxygenated and deoxygenated hemoglobin. The oximeter calculates the optical density of retinal vessels at both wavelengths. The ratio of the two densities is then approximately linearly related to the hemoglobin oxygen saturation which can then be given numerically or as a color saturation map (Figure 3) [37, 38].

Clinical studies in DR have consistently demonstrated higher retinal venous oxygen saturation in patients with DR [39–43]. At first, this may conflict the traditional concept of DR as an ischemic disease. However, the observation may be explained by (1) poor oxygen distribution to the tissue, (2) compensatory increased oxygen supply, or (3) less oxygen consumption in the retinal tissue [37]. In particular, it has been speculated that patients may have a poor oxygen distribution to the ischemic retinal tissue as given by capillary nonperfusion, arteriovenular shunting, thickening of the capillary walls, and greater affinity for oxygen in glycosylated hemoglobin [33, 37].

## 6. Fyn County Eye Study

This was a population-based cohort of patients with type 1 diabetes in Fyn County, Denmark, identified in 1973 by insulin prescriptions [44, 45]. Seven hundred twenty-seven patients were identified, and it was estimated that the patient material was more than 98% complete [44].

By March 2007, 320 patients were still alive and resident in Denmark. Of these, 208 (65.0%) agreed to participate in a clinical study [46, 47] aimed to correlate retinal noninvasive structural markers to long-term micro- and macrovascular complications. Median age and duration of diabetes were 58.7 and 43 years, respectively, and 60.0% were men.

Nine mydriatic 45° color fundus fields were captured by Topcon TRC-NW6S (Topcon, Tokyo, Japan) and graded for DR according to the Early Treatment Diabetic Retinopathy Study (ETDRS) Group adaptation of the modified Airlie House classification of DR [48, 49]. IVAN was used to grade for retinal calibers [50], and the retinal vascular fractal dimension was graded by the International Retinal Imaging Software (IRIS-Fractal), which was the standard of the time [51].

Prevalences of long-term complications were 43% for PDR and 53%, 33%, and 22% for diabetic neuropathy, nephropathy, and macrovasculopathy, respectively [52]. In a multiple logistic regression model, a lower CRAE correlated independently with diabetic nephropathy (odds ratio (OR) 2.17 per standard deviation (SD) decrease in CRAE) and macrovascular disease (OR 3.17 per SD decrease in CRAE), but not with PDR or diabetic neuropathy [17]. CRVE was unrelated to all complications.

Interestingly, a different signal was found in retinal vascular fractal analysis. In a multiple logistic regression model, a lower retinal vascular fractal dimension correlated independently with PDR (OR 1.57 per SD decrease in Fd) and diabetic neuropathy (OR 1.42 per SD decrease in Fd), but not with diabetic nephropathy or macrovascular disease [53].

In conclusion, the study demonstrated that in long-term surviving type 1 diabetic patients, noninvasive retinal markers associate with intra- as well as extraocular micro- and macrovascular complications.

## 7. The Danish Cohort of Pediatric Diabetes 1987 (DCPD1987)

The DCPD1987 was initially a population-based study of children with type 1 diabetes. Approximately 75% (*n* = 720) of all Danish type 1 diabetic children aged 18 and below participated in 1987 [54–56]. At the time of the first eye examination in 1995, the cohort had been reduced to 339 participants [54]. Surprisingly, poor glycemic control was found at every stage of the study, and in 1995, many patients showed early signs of complications with some degree of DR in 60% [54, 57, 58].

In 2011, a long-term follow-up was initiated with several purposes. On one hand, the incidence and progression of microvascular complications and mortality was of interest [59, 60]. On the other hand, the study aimed to identify early retinal markers for diabetic retinopathy, nephropathy, and neuropathy. Semiautomated computer software had in the recent years made it possible to reliably measure various parameters in the retinal vascular tree. In total, 185 participants were included. The vessel analyses were performed on retinal images from the study in 1995 and linked to microvasculopathy in 2011. In multiple regression analyses, a consistent relation between narrower retinal arteriolar calibers (OR 2.96, 2.63, and 1.56 per 10 *μ*m decrease), wider retinal venular calibers (OR 1.52, 1.76, and 1.36 per 10 *μ*m increase), and lower fractal dimensions (OR 1.17, 1.40, and 1.22 per 0.01 decrease in fractal dimension) and the 16-year incidences of diabetic neuropathy, nephropathy, and proliferative retinopathy were found [61–63]. These findings could possibly be indications of a shared pathogenic pathway for microvasculopathy in diabetes mellitus.

This study has been the longest prospective study to date of both retinal vessel calibers and retinal fractal dimensions and their predictive value on diabetic microvascular complications. In addition, the study also demonstrated cross-sectional associations between increased retinal vascular branching coefficients (BC) and diabetic nephropathy (OR 3.10 for patients with increased arteriolar BC) and diabetic neuropathy (OR 2.11 for patients with increased venular BC) [64].

## 8. Retinal Vascular Calibers in Diabetic Macular Edema (DME)

The aim of this study [65] was to evaluate global and macular retinal vessel caliber changes after focal/grid laser treatment for DME in order to provide physicians with a potential tool to monitor the progress and success of DME treatment.

We included retrospectively 69 eyes from 46 patients from a photographic screening clinic. Patients had clinically significant macular edema according to the ETDRS criteria [66] and had been treated with focal/grid laser photocoagulation according to the modified ETDRS protocol [66–68]. Furthermore, retinal photos (disc- and macula-centered) should be available from within 6 months prior to laser photocoagulation and 2–12 months after.

**Figure 4:**
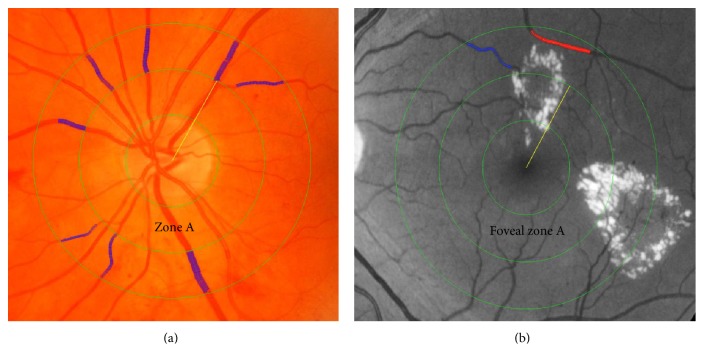
Retinal images processed by IVAN and used for vascular caliber analysis. Images are divided into zone A and zone B located 0–0.5 and 0.5–1.0 disc diameters from the center. Arterioles are marked in red and venules in blue. (a) Regular IVAN, centered at the optic disc. (b) Modified IVAN, centered at the fovea.

Computer-assisted measurements of the retinal vessels were performed by two methods: the standard IVAN method (69 eyes, 31 controls), which was used to assess the retinal vessels “globally” surrounding the optic disc margin, and a modified method m-IVAN (Figure 4) (68 eyes, 31 controls) which was used to measure macular vessels. Measurements were denoted as macular retinal arteriolar and venular equivalent (MRAE and MRVE, resp.). The modified method had a high concordance correlation coefficient (0.95 for arterioles and 0.99 for venules).

Median age was 60 years (range 29–79 years), and the overall duration of diabetes was 13 years (range 1–41 years). By the standard IVAN method, we found no significant difference in retinal vessel diameter before or after the treatment, neither in patients with DME or in untreated control eyes (Table 1).

By the m-IVAN method, we found a statistically significant decrease in vessel diameters in macular arterioles and venules after laser treatment (MRAE 73.5 *μ*m versus 72.0 *μ*m, *p* = 0.04, and MRVE 63.5 *μ*m versus 62.4 *μ*m, *p* = 0.02, resp.). In contrast, the modified analysis of the 32 untreated fellow eyes, serving as controls, showed no significant change in either arteriolar or venular diameter after laser treatment. There was no association between the type of edema (focal, *n* = 36, or diffuse, *n* = 31) and vascular caliber changes after laser treatment (data not shown).

The narrowing of the macular arterioles and venules may reflect the autoregulatory reduced blood flow caused by partial destruction of retinal tissue in accordance with the oxygen theory suggested by Stefansson et al. [35].

## 9. Noninvasive Retinal Markers of Treatment Outcome in PDR

“The individually-marked panretinal laser photocoagulation for proliferative diabetic retinopathy study” (IMPETUS 2018) has been designed to identify the threshold level of progression of PDR after panretinal photocoagulation (PRP). In particular, the study addresses noninvasive structural and metabolic markers of treatment outcome in order to archive sufficient treatment but avoid side effects like visual field loss, night blindness, and DME as caused by excessive PRP.

This is an ongoing 6-month prospective study with a cohort of treatment-naïve patients with PDR referred for PRP treatment at the Department of Ophthalmology, Odense University Hospital. We performed an interventional study with PRP as delivered by a navigated laser system (NAVILAS®, OD-OS GmbH, Berlin, Germany). Wide-field fluorescein angiography (Optomap, Optos PLC., Dunfermline, Scotland, UK), optical coherence tomography (OCT) and fundus imaging (3D OCT-2000 Spectral Domain OCT, Topcon, Tokyo, Japan), and retinal oximetry (Oxymap model T1; Oxymap, Reykjavik, Iceland) were performed at baseline and three and six months after PRP. IVAN and SIVA were used to measure retinal vascular calibers, fractal dimension, tortuosity, length-diameter ratio, and branching coefficients. At months 3 and 6, wide-field fluorescein angiography was used to evaluate PDR activity and patients were dichotomized according to progression or nonprogression of PDR.

We included 65 eyes in this study. Median age and duration of diabetes were 53.2 and 20 years, respectively, and 67% were male. At present, all clinical examinations have been concluded and the initial results have been reported.

At baseline, patients in the two groups did not differ according to age, duration of diabetes, HbA1c, blood pressure, total laser energy delivered, retinal oxygen saturation, or retinal vascular calibers. However, eyes with progression of PDR at month 3 developed a higher retinal venular oxygen saturation than those in patients without progression (+4.1% versus −1.8%, *p* = 0.02) [69]. In a multiple logistic regression analysis, each 1.0%-point increment in retinal venular oxygen saturation independently associated with a 30% higher risk of PDR progression at month 3 (*p* = 0.02) but not at month 6. These data align with prior studies that associate advanced DR with a higher retinal venular oxygen saturation [33]. We demonstrated that a postlaser decrement in retinal venular oxygen saturation indicates a favorable treatment outcome. Hence, we conclude that retinal oximetry may serve as a breaking-the-wave indicator of successful PRP.

Interestingly, the retinal calibers also correlated with treatment outcome. From baseline to month 3, the retinal arteriolar calibers decreased by 5.9 *μ*m (*p* < 0.01) in patients with progression of PDR, and likewise, the retinal venular calibers decreased by 4.7 *μ*m (*p* = 0.03) in patients with nonprogression of PDR [70]. These data support the findings from DCPD1987 [63] and others [71] which indicate that lower retinal arteriolar calibers and higher retinal venular calibers link to microvascular dysfunction in DR. Like in retinal vascular oxygen saturation measurements, this is likely to be a direct marker of treatment outcome after PRP.

## 10. Noninvasive Retinal Markers of Treatment Outcome in DME

It was established by the ETDRS that focal/grid laser photocoagulation reduces the risk of visual loss in patients with DME but with a small likelihood of visual improvement [72]. In recent years, intravitreal inhibitory vascular endothelial growth factor (VEGF) agents like bevacizumab [73], ranibizumab [74], and aflibercept [75] have consistently demonstrated efficacy and visual improvement in DME treatment. However, a high number of injections are needed to achieve sustainable visual improvement, and the burden of intravitreal injections is a substantial concern for both patients and health care systems.

Modern navigated retinal photocoagulation enables clinicians to preplan and deliver planned spots in an automatic mode as adjunctive therapy to intravitreal anti-VEGF. This led to the observation of a reduced need for intravitreal injections of bevacizumab [76] and ranibizumab [77] with a similar improvement in best-corrected visual acuity (BCVA).

The “aflibercept and navigated versus conventional laser in diabetic macular edema study” (ADDENDUM) is an ongoing, randomized 12-month prospective 1 : 1 study of patients with DME that aims to examine the treatment response of intravitreal aflibercept and navigated retinal photocoagulation (NAVILAS OD-OS GmbH, Teltow, Germany) as compared to intravitreal aflibercept and conventional retinal photocoagulation (PASCAL Photocoagulator, OptiMedica, Santa Clara, California) [78]. Based on previous work, we aim to include a minimum of 48 diabetic patients with clinical significant macular edema from the Region of Southern Denmark. Patients are randomized at baseline and receive a loading dose of three monthly intravitreal injections of aflibercept followed by central retinal photocoagulation at month 3. From month 4, patients are followed monthly and additional injections are given pro re nata.

In addition, we wish to identify noninvasive retinal markers for successful treatment outcome, and we hypothesize that treatment leads to lower retinal vascular diameters, lower retinal fractal dimensions, and higher retinal oxygen saturation in patients with a successful treatment outcome.

## 11. Conclusion

Diabetic retinopathy may lead to sight-threatening end-stage complications like DME or PDR, but so far, it has been difficult to predict patients at high risk of progression. Likewise, it is difficult to individualize treatment, and patients are often treated in a one-size-fits-all approach.

Retinal markers of structure and metabolism offer detailed and noninvasive information of the retinal vasculature. In our research group, we have strived to advance this field of research from bench to bedside. Firstly, we have demonstrated cross-sectional association between retinal calibers and fractal dimension with diabetic micro- and macrovascular complications [17, 53]. Secondly, we have established predictive values of these structural makers of long-term complications in a 16-year prospective study [61, 63] and of macular changes in patients treated for DME [65]. Finally, we have demonstrated that changes in retinal oxygen saturation and retinal vascular calibers differ according to treatment outcome in patients with PDR [69, 70], and we have a similar ongoing study in DME [78].

Diabetes has become a global epidemic. This causes an increasing burden on the health care society. Screening for DR has often been offered annually for most patients, but flexible, individualized screening intervals may reduce the number of unnecessary screening episodes for most patients [79, 80]. Given that glycemic dysregulation only explains a small proportion of the risk or DR-progression [4], additional risk markers are important. We have demonstrated that retinal arteriolar narrowing, venular dilation, and decrement in fractal dimension all independently predict long-term development of diabetic nephropathy, diabetic neuropathy, and PDR [61, 63]. A potential use of this could be automated software combining these measurements with grading of vascular DR lesions which may lead to individualized risk stratification outcomes.

There is a substantial phenotype difference in patients treated for DME and PDR. With that in mind, individualized treatment is a tempting approach, but so far, it has been difficult to make early posttreatment stratification according to treatment response. We have addressed this issue in prospective clinical trials in DME and PDR [69, 70, 78], and so far, we have demonstrated that successful treatment outcome after panretinal photocoagulation (PRP) in PDR is associated with a lower postlaser retinal oxygen saturation. On the other hand, patients with an increasing retinal venous oxygen saturation of at least 3.0% three months after PRP had a 4.0 times increased risk of progression in PDR [69]. Others have demonstrated that increasing levels of DR associate with higher retinal venous oxygen saturation [39], and, thus, it is plausible that successful treatment is able to break this dysfunctional pattern, presumably by lowering the intravascular oxygen saturation and increasing the metabolic supply to the retinal tissue.

It is a general limitation of the abovementioned studies that the structural and metabolic state of the retinal vasculature may fluctuate during many years of disease in reflection to the development of DR. This can be difficult to assess on an individualized level with only a few points of measurement captured during the years. In addition, the study on retinal vascular calibers in diabetic macular edema was limited by a relatively low number of patients, and the study was not adjusted for blood pressure, which may have confounded the results.

Diabetic retinopathy is a complex retinal disease influenced by several local and systemic factors. Noninvasive markers like retinal vascular calibers, fractals, and oxygen saturation offer additional information about the morphology and function of the retina. Likewise, novel potential biomarkers like OCT angiography may provide valuable information if cross-sectional associations with diabetes [81] and DR [82] can be confirmed in prospective studies. Repeatedly individualized measurements offer valuable information regarding the risk of progression and response to treatment. Upcoming studies should pursue this issue even further to make this an integrated part of the clinical risk assessment at our and other departments treating patients with DR.

## Figures and Tables

**Table 1:** Measurements of global and macular retinal vascular diameter.

Method	Measurement	Vascular diameter before laser treatment (*μ*m) ± SD	Vascular diameter after laser treatment (*μ*m) ± SD	*P* value
IVAN	CRAE	140.3 ± 13.2	139.3 ± 13.1	0.44
	CRVE	214.1 ± 23.6	213.1 ± 22.8	0.40
	Control CRAE	141.5 ± 14	138.6 ± 15.8	0.26
	Control CRVE	213.1 ± 24.2	214.0 ± 25.0	0.84

Modified IVAN	Macular arterioles	73.5 ± 11.2	72.0 ± 10.7	0.04^∗^
	Macular venules	63.5 ± 17.8	62.4 ± 17.6	0.02^∗^
	Control macular arterioles	71.7 ± 11.9	71.8 ± 12.2	0.89
	Control macular venules	62.0 ± 15.5	61.2 ± 15.7	0.10

Data are presented as mean with standard deviation (SD). Vessel diameters are compared before and after laser treatment with the Wilcoxon signed-rank test. ^∗^Statistically significant. CRAE: central retinal arteriolar equivalent; CRVE: central retinal venular equivalent; m-IVAN: modified IVAN; MRAE: macular retinal arteriolar equivalent; MRVE: macular retinal venular equivalent [65].
